# Examining the variation in GPs’ referral practice: a cross-sectional study of GPs’ reasons for referral

**DOI:** 10.3399/bjgp14X680521

**Published:** 2014-06-30

**Authors:** Unni Ringberg, Nils Fleten, Olav Helge Førde

**Affiliations:** Faculty of Health Sciences, UiT The Arctic University of Norway, Tromsø, Norway.; Faculty of Health Sciences, UiT The Arctic University of Norway, Tromsø, Norway.; Faculty of Health Sciences, UiT The Arctic University of Norway, Tromsø, Norway.

**Keywords:** general practice, patient preference, referral, uncertainty

## Abstract

**Background:**

There is a large variation in referral rates to secondary care among GPs, which is partly unexplained.

**Aim:**

To explore associations between reasons for referral to secondary care and patient, GP, and healthcare characteristics.

**Design and setting:**

A cross-sectional study in Northern Norway.

**Method:**

Data were derived from 44 (42%) of 104 randomly selected GPs between 2008 and 2010. GPs scored the relevance of nine predefined reasons for 595 referrals from 4350 consecutive consultations on a four-level categorical scale. Associations were examined by multivariable ordered and multivariable multilevel logistic regression analyses.

**Results:**

Medical necessity was assessed as a relevant reason in 93% of the referrals, 43.7% by patient preference, 27.5% to avoid overlooking anything, and 14.6% to reassure the patient. The higher the referral rates, the more frequently the GPs referred to avoid overlooking anything. Female GPs referred to reassure the patient and due to perceived deficient medical knowledge significantly more often than male GPs. However, perceived easy accessibility of specialists was significantly less frequently given as a reason for referral by female GPs compared with male GPs. When the GPs scored the referrals to be of lesser medical necessity, male GPs referred significantly more frequently than female GPs to reassure the patient due to patient preference and perceived deficient medical knowledge.

**Conclusion:**

There are striking differences in reasons for referral between Norwegian male and female GPs and between GPs with high and low referral rates, which reflects difficulties in handling professional uncertainty. Referring to reassure the patients, especially when referrals are less medically necessary, may reflect consideration and acquiescence towards the patients.

## INTRODUCTION

The large variation in referral rates to secondary care among GPs is partly unexplained.^1^ Patients’ age, sex, and morbidity explain less than 40% of the variation, and practice and GP characteristics less than 10%.^2^ Intrinsic psychological variables at GP level are important, but the extent of the contribution is not known.

The decision-making process for referrals is complex; increased consumerism in health care^3^ and increased legal rights of patients may increase patients’ preference for referral. In many countries, GPs are gatekeepers whose job is to manage the demand for secondary care. The decision to refer should be based on the patient’s medical condition, needs, and an assessment of the optimal level of health care. The patient should be referred at the right time after adequate pre-referral management, and after an appropriate process that also takes the patient’s wishes into account.^4^ Referrals may have several purposes, including:

- establishing a diagnosis and/or treatment plan;
- getting advice on management; or
- reassuring the GP or the patient.

The decision to refer is influenced by several non-medical factors.^5^,^6^ How GPs handle professional uncertainty is important in their decision-making process regarding referrals;^7^,^8^ and patient expectations,^9^ patient pressure,^10^,^11^ and perceived patient pressure^12^ also strongly affect GPs’ referral behaviour. Shared decision making has long been the ideal model, and Carlsen *et al* found that congruence in attitudes between GPs and patients lowered the referral rate.^13^ Little *et al* observed that referrals were scarcer if patients felt they had a personal relationship with their doctor.^14^

The main aim of this study was to explore associations between reasons for referral to secondary care, and patient, GP, and healthcare characteristics.

## METHOD

### Recruitment

Power calculation indicated a need for approximately 2500 consultations in each subgroup to detect a 25% difference in referral rates (α = 0.05, β = 0.8). Of all 476 GPs (lists) in Northern Norway 88 GPs were excluded, due to the incompatibility of electronic patient records (EPR) with the electronic questionnaire (*n* = 44), vacancy (*n* = 35), the two practices housing three GPs participating in piloting (*n* = 8), and one GP practising without EPR. Assuming there would be a response rate of 50%, a random sample of 104 of the 388 eligible GPs were invited to participate in the study. Background information on patients, GPs, their practice type (private practitioners or salaried), the municipalities, and the healthcare characteristics were collected from the GPs, the Northern Norway Regional Health Authority, and Statistics Norway. Further information on recruitment, data collection, background information, and calculation of referral rates, has been published elsewhere.^15^

**How this fits in**

The large variation in referral rates to secondary care among GPs is partly unexplained. This study scrutinises GPs’ reasons for referral at the time of referral, which differs methodologically from most comparable surveys. This study confirms previous research that part of the variation in referral rates reflects how GPs handle professional uncertainty and patient preference. Graduate and postgraduate medical education should incorporate medical decision making in the curriculum to a larger extent and focus more on how to enable GPs to handle professional uncertainty and also shared decision making. This may contribute to decreasing unwarranted variation in clinical practice.

### Data collection

Each GP completed 100 electronic questionnaires from 100 consecutive consultations; the questionnaire appeared on the GP’s computer when they closed the EPR for each patient. In the questionnaires, the GP recorded:

- whether the issue of referral was introduced during the consultation;
- if so, who introduced the issue; and
- whether the patient was referred to secondary care (somatic and psychiatric hospital outpatient services, private secondary care specialists, and/or hospital admissions) and/or radiological examination.

If the patient was referred to secondary care, the GPs scored the relevance of nine predetermined reasons for referral (Box 1) on a 4-level categorical scale, ranging from ‘corresponds very well’ to ‘does not correspond’ (Table 1). The nine reasons for referral were constructed by the authors after communication and group meetings with experienced academic and non-academic GPs. No prior validation of the questionnaire was performed but the comprehensibility, suitability, and technical design were piloted with three GPs. The present study explores the consultations with registered referral to secondary care.

Box 1. **Reasons for referring patients to secondary care^a^**

**Table:** 

**Reasons for referral**	**Abbreviated reason**
I am referring the patient because his/her medical condition makes it necessary	Medically necessary
I am referring the patient because his/her medical condition is usually taken care of in secondary health care	Common practice
I am referring the patient to avoid overlooking anything	To avoid overlooking anything
I am referring the patient because I have deficient knowledge concerning the patient’s current medical problem	Perceived deficient medical knowledge
I am referring the patient to reassure him/her	To reassure the patient
I am referring the patient because he/she wanted to be referred	Patient preference
I am referring the patient as part of a social security application	Social security application
I am referring the patient to relieve my workload	To relieve workload
I am referring the patient because the relevant specialist is easily accessible (short waiting list and/or closely located)	Perceived easily accessible specialist

a Hospital admissions, hospital outpatient services and/or private secondary care specialists (somatic and psychiatric care).

**Table 1:** Distribution of GPs’ agreement with nine predetermined reasons for referral (595 referrals)

**Reasons for referral**	**Does not correspond, % (95% CI)**	**Corresponds to a limited extent, % (95% CI)**	**Corresponds fairly well, % (95% CI)**	**Corresponds very well, % (95% CI)**
Medically necessary	0.8 (0.3 to 2.0)	6.2 (4.4 to 8.5)	28.6 (25 to 32.4)	64.4 (60.4 to 68.2)
Common practice	6.5 (4.7 to 8.9)	8.1 (6.0 to 10.6)	34.3 (30.5 to 38.3)	51.1 (47.0 to 55.2)
To avoid overlooking anything	50.8 (46.7 to 54.9)	21.7 (18.4 to 25.2)	18.1 (15.1 to 21.5)	9.4 (7.2 to 12.1)
Perceived deficient medical knowledge	51.6 (47.5 to 55.7)	27.2 (23.7 to 31.0)	15.8 (13.0 to 19.0)	5.4 (3.7 to 7.5)
To reassure the patient	63.7 (59.7 to 67.6)	21.7 (18.4 to 25.2)	10.7 (8.4 to 13.5)	3.9 (2.5 to 5.7)
Patient preference	40.8 (36.9 to 44.9)	15.5 (12.7 to 18.6)	22.4 (19.1 to 25.9)	21.3 (18.1 to 24.9)
Social security application	98.8 (97.6 to 99.5)	0.7 (0.2 to 1.7)	0.2 (0.004 to 0.9)	0.3 (0.04 to 1.2)
To relieve workload	91.1 (88.5 to 93.2)	6.9 (5.0 to 9.2)	1.5 (0.7 to 2.9)	0.5 (0.1 to 1.5)
Perceived easily accessible specialist	75.3 (71.6 to 78.7)	11.8 (9.3 to 14.6)	8.7 (6.6 to 11.3)	4.2 (2.7 to 6.1)

### Statistical analyses

All analyses were performed with Stata (version 13.0). Independence of the reasons for referral was assessed by Spearman rank-order correlation analyses. Two reasons for referral — ‘social security application’ and ‘to relieve workload’ — were dropped from analyses because some of the levels of scores contained fewer than four observations. Multivariable ordered logistic regression analyses, with calculations of standard errors that allowed for clustering at the GP level, were used to explore the association between scores for each reason for referral, and patient age, patient sex, GP age, GP sex, speciality in family medicine, practice type, travel time to nearest hospital, country where medical degree was obtained, and GPs’ referral rates.

One analysis was done for each reason for referral, checking that the assumption of proportional odds was met. Backwards elimination of variables was done when necessary to get statistically significant models. The command Gologit2^16^ was used to analyse the association between the scores for the reason ‘to avoid overlooking anything’ and background variables, because the assumption of proportional odds was not met for the variable ‘GPs’ referral rate’. Gologit2 performs generalised logistic regression for ordinal dependent variables; it can also estimate the partial proportional odds model when some variables do not meet the assumption of proportional odds, as was the case here. Interaction between patient and/or GP characteristics was tested in all analyses of the different reasons for referral. Likewise, interaction between dichotomised scores of medical necessity and GP’s characteristics on the other reasons for referral was also tested.

Multivariable multilevel logistic regression, allowing for clustering at the GP level, was used to explore the association between dichotomised scores of medical necessity of the referral, and dichotomised scores of the other reasons for referral. On an *a posteriori* basis, the reason ‘medically necessary’ was dichotomised by contrasting the three lowest agreement levels, which implied doubt, with the highest agreement level. The other reasons were dichotomised by merging the two highest and the two lowest agreement levels, respectively.

## RESULTS

After 104 GPs were contacted and sent four reminders, a total of 46 GPs agreed to participate in the study. Of these, 44 GPs in 22 practices completed the survey, yielding a response rate of 42%. The responders did not differ from the non-responders with regard to sex and mean list size, but they were a little younger (mean age 50 years versus 45 years among non-responders), more were specialists in family medicine (50% versus 40% among non-responders), more practised closer to hospitals, more were private practitioners, and more had their medical degree from Norway.^15^ Data on a total of 4350 consultations were collected between November 2008 and September 2010. Because of mainly technical reasons two GPs completed 72 and 74 questionnaires, respectively, and four GPs completed 101 questionnaires each, resulting in 50 missing questionnaires. A total of 595 consultations included a registered referral to secondary care.

### Distribution of reasons for referral

The reasons for referral (as the sum of the two highest agreement levels) were given as patient preference in 43.7% of the referrals, to avoid overlooking anything in 27.5% of the referrals, to reassure the patient in 14.6%, and because secondary care specialists were perceived easily accessible in 12.9% (Table 1). In total, 93.0% of the patients were referred due to medical necessity.

### Referral rates

GPs with referral rates in the highest quartile (high referrers) referred more frequently because secondary care specialists were perceived easily accessible (per 1% increased referral rate: odds ratio [OR] 1.10, 95% confidence interval [CI] = 0.997 to 1.22, *P* = 0.056) and significantly more frequently to avoid overlooking anything (per 1% increased referral rate: OR = 1.06) when comparing the three highest agreement levels with the lowest (Table 2).

If the two highest agreement levels in Table 3 are summed, high referrers reported that an adjusted percentage of 32.7 of their referrals were effectuated to avoid overlooking anything, contrasted by GPs with referral rates in the lowest quartile (low referrers) who reported this in only 11.9% of their referrals. High referrers reported that 17.8% of their referrals were carried out because specialists were perceived easily accessible; low referrers stated this in 5.3% of their referrals.

**Table 2:** Associations between reasons for referral and patient, GP, and healthcare characteristics (595 referrals)

**Reason for referral**	**GP age per 10 years, OR (95% CI)**	**GP sex Male = 0 Female = 1, OR (95% CI)**	**Patient age per 10 years, OR (95% CI)**	**Patient sex Male = 0 Female = 1, OR (95% CI)**	**Speciality in family medicine No = 0 Yes = 1, OR (95% CI)**	**Travel time to nearest hospital per 60 mins, OR (95% CI)**	**GPs’ referral rate per 1% increase, OR (95% CI)**
Medically necessary^a^	1.06 (0.77 to 1.45)	1.26 (0.74 to 2.15)	1.10^b^ (1.02 to 1.18)	0.70 (0.48 to 1.02)	0.73 (0.34 to 1.58)	1.20 (0.95 to 1.53)	0.97 (0.92 to 1.02)
To avoid overlooking anything^c^	–	0.90 (0.50 to 1.64)	–	1.10 (0.83 to 1.48)	–	1.20 (0.87 to 1.66)	1.06^b,d^ (1.01 to 1.11)
Perceived deficient medical knowledge^a^	0.85 (0.68 to 1.08)	2.22^b^ (1.47 to 3.36)	0.94 (0.87 to 1.02)	0.82 (0.58 to 1.16)	0.997 (0.59 to 1.68)	1.22 (0.97 to 1.53)	1.05 (0.99 to 1.11)
To reassure the patient^a^	0.87 (0.63 to 1.20)	1.97^b^ (1.11 to 3.50)	1.04 (0.96 to 1.12)	1.06 (0.74 to 1.51)	1.17 (0.60 to 2.27)	1.25 (0.90 to 1.74)	1.03 (0.97 to 1.09)
Patient preference^a^	0.54^b^ (0.40 to 0.74)	1.36 (0.71 to 2.60)	1.01 (0.95 to 1.08)	1.10 (0.80 to 1.52)	3.18^b^ (1.54 to 6.57)	1.46^b^ (1.002 to 1.90)	1.03 (0.96 to 1.11)
Perceived easily accessible specialist	0.74 (0.50 to 1.11)	0.29^b^ (0.09 to 0.995)	–	–	–	–	1.10^e^ (0.997 to 1.22)

a Analysed by multivariable ordered logistic regression, reporting standard errors that allowed for clustering at GP level. Adjusted for patient age, patient sex, GP age, GP sex, speciality in family medicine, practice type, travel time to nearest hospital, country where medical degree was obtained, and GPs’ referral rates in analyses of ‘perceived deficient medical knowledge’, ‘to reassure the patient’, and ‘patient preference’. Adjusted for the former background variables except practice type in ‘medically necessary’, and adjusted for background variables presented in the row for ‘perceived easily accessible specialist’ (see Method).

b P*<0.05.*

c Analysed by multivariable ordered logistic regression, by the command Gologit2, reporting standard errors that allowed for clustering at GP level, and adjusted for background variables presented in the row (see Method).

d OR = 1.06 comparing the three highest agreement levels (2+3+4) with the lowest level (0). OR = 1.009 (95% CI = 0.97 to 1.05) level 1+2 versus 3+4, and OR = 1.004 (95% CI = 0.94 to 1.08) level 1+2+3 versus 4.

e P *= 0.056. OR = odds ratio.*

**Table 3:** Multivariable adjusted^a^ distribution of percentages of GPs’ agreement with reasons for referral^b^ by quartiles^c^ of GPs’ referral rates and sex^d^

**Reasons for referral**	**Does not correspond**	**Corresponds to a limited extent**	**Corresponds fairly well**	**Corresponds very well**

	**%, adjusted**	**%, adjusted**	**%, adjusted**	**%, adjusted**
**To avoid overlooking anything**				
Highest quartile of referral rates	37.1	30.3	20.4	12.3
Lowest quartile of referral rates	78.9	9.9	8.8	3.1

**Perceived deficient medical knowledge**				
Female GPs	39.0	30.3	19.5	6.5
Male GPs	59.0	24.4	12.3	2.3

**To reassure the patient**				
Female GPs	53.7	25.9	12.3	4.9
Male GPs	70.3	17.8	8.5	1.7

**Perceived easily accessible specialist**				
Highest quartile of referral rates	68.1	11.6	11.0	6.8
Lowest quartile of referral rates	84.1	9.8	4.3	1.0
Female GPs	86.9	9.5	2.3	1.8
Male GPs	69.5	12.9	9.2	4.4

a *For each reason for referral adjustments are made for the same background variables as in *Table 2.

b *Reasons for referral with significantly different distribution with respect to GPs’ referral rates and/or GP sex (see *Table 2).

c Quartiles of referral rates per 100 consultations: lowest quartile: referral rates <10% (86 referrals), highest quartile: referral rates >16 % (219 referrals).

d 219 referrals from female GPs and 376 from male GPs.

### GP sex

Compared with male GPs, female GPs significantly more frequently referred because of perceived deficient medical knowledge and to reassure the patient (OR = 2.22 and 1.97, respectively) (Table 2). However, perceived easy accessibility of specialists was significantly less frequently given as reasons for referral by female GPs (OR = 0.29).

When the two highest agreement levels in Table 3 are summed female GPs referred due to perceived deficient medical knowledge in 26.0% of referrals compared to 14.6% among males. In addition, they referred to reassure the patients in 17.2% of their referrals, compared with 10.2% of male GPs’ referrals. Conversely, 4.1% of referrals from female GPs were carried out because specialists were perceived easily accessible, versus 13.6% of male GPs’ referrals.

### GP age and healthcare organisation

The increasing age of the GPs significantly reduced patient preference as reasons for referral (10-year OR = 0.54, Table 2). Referrals to private specialists were significantly more often explained by perceived easy access to specialists compared with hospital outpatient services (OR = 5.6; 95% CI = 2.9 to 10.9, data not shown).

### Medically necessary and other reasons for referral

There was significant interaction between GP sex and dichotomised scores for medical necessity on the reasons ‘to reassure the patient’ and ‘perceived deficient medical knowledge’. The results were, therefore, stratified by sex (Table 4). When male GPs considered the referrals to be less medically necessary, merging the three lowest agreement levels, they significantly more frequently referred due to perceived deficient medical knowledge, to reassure the patient and due to patient preference (OR = 4.06, 13.44 and 3.28, respectively), which did not apply to female GPs. In the less medically necessary referrals male GPs that scored 28.2% were motivated to reassure the patient, compared with 2.9% in the medically necessary referrals. In contrast, female GP scored to reassure the patient in 26.0% versus 15.5%.

**Table 4:** Associations^a^ between medical necessity and other reasons for referral, stratified by GP sex

**Reasons for referral**	**Referrals by male GPs**			**Referrals by female GPs**			**All referrals**
	**Medically necessary^b^, = 0**	**Less medically necessary^c^, = 1**	**OR (95% CI)**	**Medically necessary^b^, = 0**	**Less medically necessary^c^, = 1**	**OR (95% CI)**	**OR^a^ (95% CI)**
	***n* = 241**	***n* = 135**	***n* = 376**	***n* = 142**	***n* = 77**	***n* = 219**	***n* = 595**
**Perceived deficient medical knowledge**			4.06^d^ (1.89 to 8.72)			1.25 (0.60 to 2.60)	2.24^d^ (1.34 to 3.76)
Agree, %	10.0	27.4		28.9	31.2		
Disagree, %	90.0	72.6		71.1	68.8		

**To reassure the patient**			13.44^d^ (4.77 to 37.88)			1.35 (0.49 to3.70)	4.12^d^ (2.23 to 7.63)
Agree, %	2.9	28.2		15.5	26.0		
Disagree, %	97.1	71.8		84.5	74.0		

**Patient preference**			3.28^d^ (1.81to 5.96)			2.08 (0.97 to 4.49)	2.66^d^ (1.69 to 4.19)
Agree, %	29.1	61.5		46.5	53.3		
Disagree, %	70.9	38.5		53.5	46.8		

a Analysed by multivariable multilevel logistic regression, allowing for clustering at GP level, adjusted for the reasons ‘perceived deficient medical knowledge’, ‘to reassure the patient’, ‘patient preference’, and ‘common practice’.

b The highest agreement level of medically necessary: ‘corresponds very well.

c The three lowest agreement levels of medically necessary ‘does not correspond’, ‘corresponds to a limited extent’, and ‘corresponds fairly well’ were merged.

d P*<0.05. OR = odds ratio.*

## DISCUSSION

### Summary

Although the vast majority of referrals were scored as medically necessary, patients’ preference for referral was concurrently given as a reason in 43.7%, and 27.5% of the referrals were effectuated to avoid overlooking anything. Female GPs referred more often to reassure the patient and due to perceived deficient medical knowledge than males, and high referrers referred more often to avoid overlooking anything than low referrers. Compared to their counterparts, female GPs and low referrers referred less frequently due to accessibility of secondary care specialists. For referrals considered to be less medically necessary, male GPs referred more often due to perceived deficient medical knowledge, to reassure patients, and due to patient preference, compared to female GPs.

The Spearman rank-order correlation analyses of the reasons for referral displayed an α of 0.55 for the reasons ‘to avoid overlooking anything’ and ‘to reassure the patient’. However, it was decided that these reasons for referral would not be combined because they give specific and different information about the decision-making process.

### Strengths and limitations

A considerable strength of this study is that the questionnaires mandatorily appeared on screen after each consultation. They were also consecutively completed immediately after the consultations, giving a representative sample of patients and referrals. The GPs scored all nine reasons almost immediately after they made the referral decision, which minimised recall bias. The referral data were collected throughout the year, and the participating GPs were all working in ordinary general practices (as opposed to academic or other special types of practice).

The predetermined reasons for referral in this study particularly explored GPs’ professional uncertainty, as well as their perception of patients’ uncertainty and preference for referral. Together, these variables constitute important motives inherent in the decision to refer. It is possible that GPs may have perceived some of the predetermined reasons for referral to be sensitive and been inclined to provide opportunistic scoring, emphasising answers that were ‘professionally correct’. However, the fact that this study used a self-administered questionnaire, and that the GPs had to respond to, and balance between, all nine reasons, probably reduced this tendency.^17^ A categorical scale with an even number of response categories was chosen; as such, GPs were offered no neutral options that they could choose.

The pop-up questionnaire was constructed for specific use in general practice and to explore parts of the GPs’ decision-making process regarding referrals. It was prepared in collaboration with experienced academic and non-academic GPs, and piloted among other GPs. Both responses from the pilot and from the participating GPs in the survey supported the assumption that it was easy to score the different reasons for referral. The authors therefore believe that the content validity of the questionnaire is satisfactory.

The response rate of 42% raises the concern of selection bias. The responders did not differ from the non-responders with regard to sex. The non-responders were older than the responders and fewer were specialists in family medicine.^15^ However, the responders were more comparable to the whole population of GPs in Northern Norway than the non-responders. This study has indications that the non-responders’ referral rates were slightly higher than the responders’ (25.6% of the list population per year to hospital outpatient clinics versus 23.4%; personal communication, Center of Clinical Documentation and Evaluation [SKDE], Northern Norway Regional Health Authority, 2011).

This may indicate that the observed referral rates in this study more probably represent an underestimation. It is not known if the non-responders would have assessed the reasons for referral differently from the responders. Most probably, the differences by GPs’ referral rate may be underestimated rather than overestimated. Altogether, the authors believe these issues vouch for a reasonable external validity.

### Comparison with existing literature

#### Professional uncertainty and accessibility to secondary care

Referring a patient to secondary care in order not to overlook anything is a common and legitimate reason for referral. However, the threshold for choosing this reason differs among GPs and demonstrates how they handle professional uncertainty.^7^ High referrers reported that about one-third of their referrals were carried out to avoid overlooking anything, compared with only 11.9% among low referrers. The results revealed a reduced tolerance for uncertainty among high referrers.

Female GPs in this study referred more often than male GPs due to perceived deficient medical knowledge. Research has shown that female physicians report more stress from uncertainty,^18^ and that referral rates are higher among primary care doctors who experience greater stress from, and have a reduced tolerance of, uncertainty.^19^,^20^ As such, this study’s findings are in accordance with the sex difference reported elsewhere.

Although not directly comparable to this study, Allison *et al* reported that, using a physician response-to-uncertainty scale, ‘each standard deviation increase in “anxiety due to uncertainty’ corresponded to a 17% increase in mean charges”.^21^ This also corresponds well with Fiscella *et al*, who reported that:

*‘After adjustment for case mix, risk-averse physicians generated higher expenditures; a one standard deviation increase in risk-aversion was associated with a 3% increase in expenditures’.*^22^

And Ghosh stated that:

*‘Most physicians, however, respond to resolving uncertainty by action, and studies have revealed that this behaviour could lead to increased hospital admission and ordering of tests’.*^7^

The observation that high referrers’ decision to refer was, to a larger extent, influenced by perceived accessibility of secondary care is in line with the concept of supply-induced demand.^23^ However, this study’s authors found nothing to explain why, among high referrers, it was mainly male GPs who referred due to the perceived accessibility of secondary care.

#### Reassuring patients and patients’ referral preference

Referrals made by female GPs were more often motivated by reassuring the patients, which may reflect a higher female sensitivity to patient preference and need for reassurance. This corresponds with the findings of a meta-analysis that showed communication among female primary care physicians was more ‘patient centred’, that females engaged ‘in significantly more active partnership behaviours’, and that their communication included ‘emotionally focused talk’.^24^ Morgan *et al* reported that there were variations in:

*‘... individual’s willingness or “resistance” to refer, reflecting differences in ... personal tolerance of uncertainty, views of patients’ “right”* [s] *to referral.’* 25

Along with fading medical paternalism, younger GPs’ referrals were more often motivated by the patients’ preference for referral.

#### Medically unnecessary referrals versus professional uncertainty and patient-related reasons for referral

As expected, the majority of referrals were characterised as medically necessary; any deviation from this would be close to admitting unprofessional practice. However, this study’s finding that those referrals that were less medically necessary were more frequently associated with reassuring the patient and due to his/her preference, has, to the authors’ knowledge, not been demonstrated previously.

The present study confirms and differentiates interpretations from methodologically different studies, using postal questionnaire,^6^,^19^ interviews,^8^ and associations between psychometric assessment of the doctors and referral rates.^21^,^22^ Therefore, part of the variation in GPs’ referral practice is related to how GPs handle professional uncertainty and to patient preference, both of which are inherent features of contemporary general practice.

### Implications for practice

For many years professional uncertainty has been proclaimed as an important influential factor in medical decision making. Nevertheless, many clinicians are unaware of the importance of this element and how it influences their medical decisions.^26^

Graduate and postgraduate medical education should incorporate medical decision making in the curriculum to a larger extent, and focus on how to handle professional uncertainty and also shared decision making; to help GPs manage professional uncertainty, inherent in all medical decision making, and improve their process of decision making. This may contribute to decreasing unwarranted variation in clinical practice, particularly regarding referrals. The differences in the relative importance of reasons for referral between male and female GPs and high and low referrers are striking, and reflect difficulties in handling professional uncertainty. Referring to reassure patients, especially in less medically necessary situations, may reflect enhanced consideration and acquiescence towards those patients. Future research should focus on exploring tools that will improve the GPs’ decision making process and consequently contribute to better results regarding patient outcome, the optimal use of healthcare resources, and GPs’ job satisfaction.
